# Type I and Type II Alcoholism: An Update

**Published:** 1996

**Authors:** C. Robert Cloninger, Sören Sigvardsson, Michael Bohman

**Affiliations:** C. Robert Cloninger, M.D., is Wallace Renard Professor of Psychiatry and Genetics and director of the Center for Psychobiology of Personality at the Washington University School of Medicine, St. Louis, Missouri. Sören Sigvardsson, Ph.D., is associate professor in the Department of Social Medicine and Michael Bohman, M.D., is professor emeritus in the Department of Child and Youth Psychiatry, University of Umeå, Umeå, Sweden

**Keywords:** AOD dependence, disorder classification, Cloninger’s typology, adoption study, Sweden, comparative study, hereditary factors, environmental factors, personality trait, criminality, age

## Abstract

A commonly cited alcoholism typology, the type I-type II typology, was developed from the findings of a study of Swedish adoptees and their biological and adoptive parents. Type I alcoholism affects both men and women, requires the presence of a genetic as well as an environmental predisposition, commences later in life after years of heavy drinking, and can take on either a mild or severe form. Type II alcoholism, in contrast, affects mainly sons of male alcoholics, is influenced only weakly by environmental factors, often begins during adolescence or early adulthood, is characterized by moderate severity, and usually is associated with criminal behavior. Additional studies have demonstrated that type I and type II alcoholics also differ in characteristic personality traits (e.g., harm avoidance and novelty seeking) as well as in certain neurophysiological markers. A replication study with a second group of Swedish adoptees has confirmed many of the findings of the original adoption study.

Avast number of alcoholism^1^ typologies have been developed during the past one-and-a-half centuries. Equally diverse are the factors used to distinguish between different alcoholism subtypes within these various typologies. These factors include personality characteristics, coexisting psychiatric disorders, gender, and alcohol consumption patterns (for review, see the article by Babor, pp. 6–14.). One frequently cited typology resulted from a study of alcoholism and other relevant characteristics in a large number of Swedish adoptees and their biological and adoptive parents. This typology distinguishes alcoholics according to the inheritance patterns of their alcoholism (i.e., whether the involved genes are passed on primarily from father to son or from both parents to both sons and daughters) and the relative contributions of genetic and environmental factors to the individual’s susceptibility for developing the disease (Cloninger et al. 1981). The two subtypes identified in this typology are called type I (milieu-limited) and type II (male-limited) alcoholism.

This article reviews the findings of the Stockholm adoption study on which this typology was based and summarizes characteristics of both type I and type II alcoholics as identified in these and subsequent analyses. Finally, the article presents data from a recent replication of the Stockholm adoption study in a different population of Swedish adoptees.

## The Stockholm Adoption Study

For many years scientists have known that alcoholism tends to run in families, suggesting that inherited characteristics play a role in its development (Amark 1951). Genetic factors alone, however, cannot determine who becomes an alcoholic: Environmental factors also play a role in establishing a person’s susceptibility to alcoholism (Kaij 1960). To investigate the relative contributions of genetic and environmental factors to the development of alcoholism in more detail, Cloninger and colleagues (1981) studied a sample of 862 male Swedish adoptees. This study population comprised all sons born to single women in Stockholm, Sweden, between 1930 and 1949 whose fathers were known and who were adopted by nonrelatives at an early age. The investigation also included the adoptees’ biological and adoptive parents. The researchers followed both the adoptees and their parents over several decades. At the time of the last data collection, the adoptees ranged in age from 23 to 43.

In collecting the study data, the researchers made use of several characteristics of the Swedish social system that allow extensive data collection. For example, child welfare agencies record the ages, occupations, and residences of the biological and adoptive parents; the criminal registry contains records of criminal convictions; local agencies of the National Health Insurance chronicle medical diagnoses and hospitalizations; and hospital records contain information about treatment for psychiatric disorders. In addition, extensive records exist documenting a person’s history of alcohol abuse. Each community has a so-called temperance board that enforces social sanctions for alcohol abuse (e.g., imposes fines) and orders and supervises alcoholism treatment. Thus, temperance board registries document how often a person has been cited or treated for alcohol abuse. Using these data, the researchers established detailed histories for the adoptees, their biological parents, and their adoptive parents that contained information about each subject’s socioeconomic status, medical history, alcohol abuse history, and contacts with the criminal justice system.

### Findings of the Stockholm Adoption Study

The researchers first investigated whether alcohol abuse in either the biological or adoptive parents increased the risk for alcohol abuse in the adopted-away sons (reviewed in Sigvardsson et al. in press). The study found that alcoholism in at least one birth parent increased the son’s risk of abusing alcohol (table 1), whereas alcoholism in the adoptive parents did not.

More detailed analyses of characteristics of the biological parents and their adopted-away sons demonstrated that the adoptees fell into two groups with respect to their alcohol abuse patterns and birth parent characteristics predisposing them to alcoholism (Cloninger et al. 1981). The first group—type I alcoholics—included adoptees with mild or, in some cases, severe alcohol abuse. (The severity of alcohol abuse was determined by the subject’s number of registrations with the temperance board and whether he had undergone alcoholism treatment.) A genetic predisposition (i.e., the presence of alcoholism in one of the birth parents) contributed only slightly to this type of alcoholism. Any alcohol abuse in the birth parents usually was mild, required no treatment, commenced during adulthood, and was not associated with significant criminality. The adoptive environment, especially in families in which the father held a low occupational status (i.e., was an unskilled laborer), also contributed to the frequency and severity of alcohol abuse in adoptees with type I alcoholism.

The second group of alcohol-abusing adoptees, called type II alcoholics, was characterized by more moderate alcohol abuse, compared with type I alcoholics. Predisposing factors for this type of alcoholism generally included severe alcoholism in the birth father that required extensive treatment and frequently was associated with severe criminality. Both the father’s alcoholism and criminality often commenced during adolescence. The birth mothers of type II alcoholics generally did not abuse alcohol. The adoptive environment appeared to contribute to the severity of type II alcoholism but did not affect its frequency. Although type II alcoholism overall was much less common among the male adoptees than type I alcoholism, men who were genetically predisposed to type II alcoholism were at a significantly higher risk of becoming alcoholic themselves than men with a genetic or environmental predisposition to type I alcoholism.

A companion study using the same Swedish population investigated the susceptibility to alcoholism in 913 female adoptees with the same characteristics as the male adoptees described previously (Bohman et al. 1981). The study showed that, in general, alcohol abuse in the birth fathers only slightly increased the risk for alcohol abuse in adopted-away daughters, whereas alcohol abuse in the birth mothers or in both parents increased the risk significantly (table 1). Both alcoholic fathers and mothers with certain characteristics (e.g., mild alcohol abuse, minimal criminality, and low occupational status) increased their adopted daughters’ risk of alcohol abuse. Environmental factors (e.g., occupational status of the adoptive father) also played a small but significant role in determining the adopted daughters’ risk for alcoholism. However, as with male adoptees, alcohol abuse in the adoptive parents did not influence the female adoptees’ risk for alcohol abuse. In summary, the pattern of inheritance of alcohol abuse among female adoptees corresponded to the pattern observed in type I male adoptees, indicating that type I alcoholism can affect both men and women, whereas type II alcoholism is primarily limited to men. (In later studies, however, a certain proportion of female alcoholics also fit the personality profile associated with type II alcoholism.)

**Figure:**
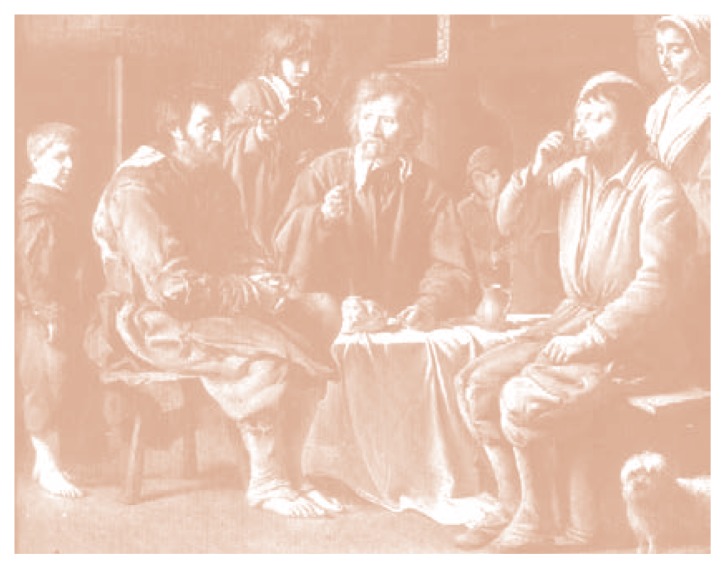
Familial alcoholism illustrated in “The Peasants Supper,” 1642, by Les Fréres Le Nain. Reproduced with permission from the Musée du Louvre. © des Musées Nationaux, Agence Photo RMN.

The studies’ conclusion about the existence of two alcoholic subtypes generated widespread interest and stimulated numerous additional studies, because the study had several important strengths compared with previous adoption studies. First, it included a large number of subjects who had not been selected for any specific characteristics other than being adoptees. Second, the adoptees and both their biological and adoptive parents were monitored for alcohol abuse and other related characteristics during their entire lifetimes. Previous studies usually had evaluated the birth parents only up to the time of adoption, when alcoholism and other behaviors may not yet have developed. In addition, those studies frequently lacked information about the birth fathers. Third, the study had used multiple data sources and efficient statistical methods to classify the subjects’ alcohol abuse and determine relevant characteristics of the adoptees’ genetic and environmental backgrounds.

## Differences Between Type I and Type II Alcoholics

Since the type I-type II alcoholism classification was developed, many researchers have confirmed the findings of the original studies and have further investigated differences between the two subtypes. In these studies, the age at onset and the type of alcohol-related problems emerged as the characteristics that most readily distinguished between the two subtypes^2^ (see table 2) (Babor et al. 1992; Gilligan et al. 1988; von Knorring et al. 1987*a*). Type I alcoholism developed during adulthood and generally was characterized by binge drinking (i.e., prolonged drinking bouts with default of responsibilities), interspersed with prolonged periods of abstinence; loss of control over drinking; excessive guilt about drinking; and rapid progression from mild to severe alcohol abuse, often accompanied by the development of alcoholic liver disease. Conversely, type II alcoholism generally commenced during adolescence or early adulthood, and alcohol consumption frequently was accompanied by fighting and arrests. In addition, alcohol abuse was moderately severe and frequently required treatment, although the severity of abuse did not change over time as it did in type I alcoholism.

Other researchers detected differences between type I and type II alcoholics not only in the age at onset and the type of alcohol-related problems, but also in certain neurobiological markers. For example, several studies found that compared with type I alcoholics, type II alcoholics exhibited lower activity levels of the enzyme monoamine oxidase (MAO) (von Knorring et al. 1987*b*; Sullivan et al. 1990). MAO is involved in metabolizing certain brain chemicals (i.e., neurotransmitters) that mediate signal transmission among nerve cells. One neurotransmitter metabolized by MAO is serotonin. Accordingly, reduced MAO activity could indicate a reduced turnover of serotonin in the central nervous system (CNS) (Oreland and Shaskan 1983).

Virkkunen and Linnoila (1990) also found that type I and type II alcoholics differed in their serotonin activities in the CNS. The serotonin levels were reduced in the brains of men with early-onset alcoholism accompanied by violent behavior (i.e., type II alcoholics).

Type I and type II alcoholics also differed in their patterns of electrical brain waves as measured by an electroencephalogram (EEG). These differences existed when the subjects were resting (Cloninger 1987*a*), but also when they were exposed to certain stimuli. Uncommon stimuli interspersed among common stimuli (e.g., a rare green light among a sequence of red and yellow lights) elicit brain waves, called event-related potentials (ERP’s), that are made up of several components. A commonly studied ERP component is called P300, because it occurs about 300 milliseconds after the uncommon stimulus. Branchey and colleagues (1988) found that the P300 height (i.e., amplitude) was lower in violent alcoholics (i.e., likely corresponding to type II alcoholics) than in nonviolent alcoholics (i.e., likely corresponding to type I alcoholics). The P300 amplitude also was reduced in people at risk for type II alcoholism, such as sons of type II alcoholics who were not alcohol dependent themselves (Begleiter et al. 1987).

These neurobiological markers previously had been associated with certain stable personality traits. For example, low MAO activity was related to impulsiveness, desire to avoid monotonous tasks, extroversion, and sensation-seeking behavior (von Knorring et al. 1987*a*). Moreover, reduced P300 amplitudes appeared to reflect the subjects’ inability to distinguish between common and uncommon stimuli (Cloninger 1987*a*). Accordingly, researchers investigated whether reproducible differences in personality traits existed between type I and type II alcoholics.

These analyses identified three heritable personality traits that could describe the prototypical characteristics of both alcoholism subtypes: harm avoidance, novelty seeking, and reward dependence (Cloninger 1987*a*). The term “harm avoidance” describes whether a person is cautious, apprehensive, pessimistic, and inhibited (i.e., high harm avoidance) or confident, relaxed, optimistic, and uninhibited (i.e., low harm avoidance). People with high novelty-seeking traits are impulsive, exploratory, and distractible, whereas people with low novelty-seeking traits are rigid, reflective, and attentive to detail. Finally, a high reward dependence describes subjects who are eager to help others, emotionally dependent, sentimental, and sensitive to social cues, whereas subjects with low reward dependence are socially detached, emotionally cool, practical, and tough minded.

In initial studies, type I alcoholics frequently exhibited high harm avoidance, low novelty seeking, and high reward dependence (Cloninger 1987*a*), personality characteristics indicating high levels of anxiety. Type II alcoholics often exhibited a reverse personality profile, with low harm avoidance, high novelty seeking, and low reward dependence. This combination of traits also describes people with antisocial personality disorder (ASPD) (Cloninger 1987*b*) and is consistent with findings that type II alcoholics frequently suffer from ASPD (Gilligan et al. 1988).

The differences in personality traits between type I and type II alcoholics led to a hypothesis about the underlying motivation for alcohol consumption in the two subtypes (Cloninger 1987*a*). According to this theory, type I alcoholics experience a late onset of alcoholism because their high harm avoidance trait initially inhibits the initiation and frequency of drinking. After an extended period of socially encouraged drinking (e.g., drinking with coworkers at lunch), the risk of alcoholism increases, because the drinkers experience relief of their anxieties after alcohol consumption. For type II alcoholics, who primarily are characterized by high novelty seeking, alcohol use is motivated by the desire to induce euphoria. This desire, which also may lead to other drug abuse, generally begins during adolescence or early adulthood.

The personality traits of harm avoidance, novelty seeking, and reward dependence likely are inherited independently of each other and are influenced by three brain systems that differ in the neurotransmitters they use (Cloninger 1987*a*). For example, the brain system for novelty seeking is predominantly influenced by the neurotransmitter dopamine (Cloninger 1987*a*). Accordingly, people who have a high novelty seeking trait are expected to react strongly to the stimulation of dopamine-using (i.e., dopaminergic) nerve cells. Researchers recently confirmed this hypothesis after finding the predicted correlations between novelty seeking, plasma prolactin levels, and heritable variants of cellular components mediating dopamine’s effects (Cloninger 1996).^3^ Likewise, the serotonin-using (i.e., serotonergic) nerve cells have complex effects on behavior, including facilitating harm avoidance and social cooperation (a measure of high reward dependence) (Cloninger 1995).

These observations suggest certain patterns of neurotransmitter activity in different alcoholic subtypes. For example, people with antisocial personality traits or type II alcoholism are expected to be uncooperative and to have low serotonergic activity in the CNS. Moreover, these individuals are expected to be high in novelty seeking and, therefore, low in dopaminergic CNS activity. In contrast, type I alcoholics, who typically are high in harm avoidance and reward dependence, are likely to be high in both dopaminergic and serotonergic CNS activity (Cloninger 1995). These predictions reflect the empirical findings that type II alcoholics consistently exhibit high novelty-seeking traits and low cooperativeness; however, their levels of harm avoidance may vary.

Because the personality characteristics are inherited independently of each other, traits such as high harm avoidance and high novelty seeking are not mutually exclusive and can occur in the same person. Accordingly, type I and type II alcoholism are not discrete diseases or separate entities; instead, alcoholism in each person is the manifestation of his or her individual combination of personality traits (Sigvardsson et al. in press). Thus, the type I and type II prototypes only represent the two extremes of a continuous spectrum of manifestations of alcohol abuse.

## A Replication Study in Swedish Adoptees

Although the type I-type II distinction has become widely accepted since its inception and has stimulated a large body of research, there also has been skepticism about some of the results of the original Stockholm adoption study. For example, it seemed unlikely that one should be able to distinguish different categories of alcoholics based on the severity of their alcohol abuse (i.e., one subtype comprises alcoholics with mild or severe abuse, whereas the other subtype consists of alcoholics with moderate alcohol abuse) (Searles 1988; Littrell 1988). Similarly, it was unexpected that the genetic backgrounds of mild and severe alcohol abusers should be the same, whereas the genetic background of moderate abusers differed. Although some of these criticisms already had been addressed by analyses defining additional characteristics of type I and type II alcoholics, the Stockholm adoption study was not replicated independently until recently (Sigvardsson et al. in press). The following section summarizes the findings of this replication study, which included adoptees from Gothenburg, Sweden, and their biological and adoptive parents.

The Gothenburg study replicated the Stockholm study by including all children born to single mothers between 1930 and 1949 whose fathers were known and who were adopted by nonrelatives at an early age. Based on these criteria, the study evaluated 577 male and 660 female adoptees. The researchers also used the same sources to obtain comprehensive information about the adoptees, their biological parents, and their adoptive parents and employed the same criteria to classify the adoptees’ alcohol abuse severity. The adoptees’ genetic and environmental backgrounds were determined based on criteria that had emerged during the Stockholm study (e.g., a genetic background predisposing for type II alcoholism was characterized by the onset of recurrent alcohol abuse during adolescence and criminality in the biological father; an environmental background predisposing for severe type I alcoholism was characterized by an adoptive father with a low occupational status).

**Figure 1:**
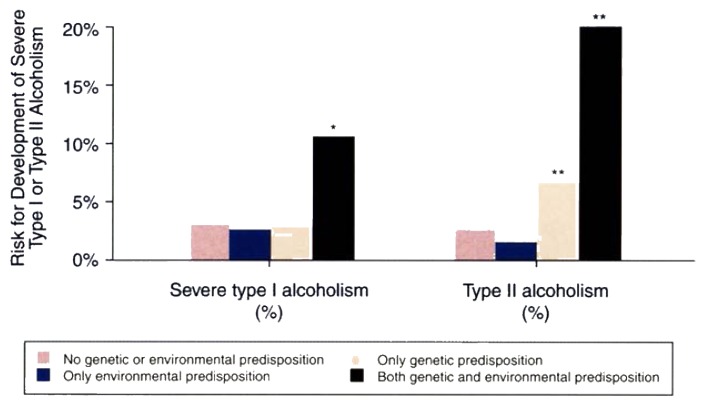
Risk for development of severe type I or type II alcoholism among adoptees from Gothenburg, Sweden, with the appropriate genetic or environmental predisposition. ^*^Significant difference in all other groups. ^**^Significant difference in adoptees without a genetic predisposition. SOURCE: Adapted from Sigvardsson et al. in press.

Using these criteria, the replication study confirmed many of the original study’s findings, as follows:

- Among male adoptees, the risk of alcohol abuse was higher in subjects with at least one alcoholic birth parent (24.1 percent) compared with subjects whose biological parents were not alcoholic (12.8 percent). Conversely, among female adoptees, alcohol abuse in the biological father did not increase the daughter’s risk for alcohol abuse. The number of female adoptees with alcoholic mothers was too small to determine whether an alcoholic birth mother increased the daughter’s risk for alcohol abuse as suggested by the Stockholm adoption study (Bohman et al. 1981).
- Male adoptees with both a genetic and an environmental background predisposing them to severe type I alcoholism had a higher risk of type I alcoholism than adoptees with no or with only one of these predispositions (figure 1).
- Male adoptees with a genetic background predisposing them to type II alcoholism had a significantly higher risk of type II alcoholism than those with no predisposition or only an environmental predisposition. The combination of both a genetic and an environmental predisposition further increased the risk for type II alcoholism (figure 1). Conversely, a genetic and/or environmental predisposition to type II alcoholism did not increase the adoptees’ risk for type I alcoholism.

However, there also were discrepancies between the findings of the Stockholm and Gothenburg studies. For example, in the original study, the risk for mild type I alcohol abuse increased in male adoptees with both a type I genetic background and an environmental background predisposing them to mild type I alcoholism. In the replication study, however, male adoptees with these characteristics only exhibited an increased risk of severe type I alcohol abuse. The reason for this discrepancy is still unknown. It is possible that mild alcohol abuse, which is defined as a single registration with the temperance board, cannot be measured reliably or is not inherited consistently. Alternatively, other inherited characteristics in the male adoptees from Gothenburg with a genetic predisposition for type I alcoholism also might predispose them to a lower occupational status, which, in turn, is associated with an increased risk for severe type I alcoholism.

## Summary

Adoption studies investigating the relative contributions of genetic and environmental factors to a person’s susceptibility to alcoholism have identified two alcoholism subtypes that differ in their inheritance patterns as well as in other characteristics. A predisposition for type I alcoholism, which affects both men and women, requires the presence of a specific genetic background as well as certain environmental factors. This alcoholism subtype is characterized by mild or severe alcohol abuse, adult onset of the disease, a loss of control over drinking, and guilt and fear about alcohol dependence. People with this alcoholism subtype generally exhibit high harm avoidance and low novelty-seeking personality traits and drink primarily to relieve anxiety. In contrast, type II alcoholism, which occurs more commonly in men than in women, primarily requires a genetic predisposition; environmental factors only play a minor role in its development. Type II alcoholism is associated with an early onset (i.e., before age 25) of both alcohol abuse and criminal behavior and an inability to abstain from alcohol. The most common personality characteristic of type II alcoholics is high novelty seeking. These people consume alcohol primarily to induce euphoria. The differences in heritable personality characteristics and the interaction of these characteristics with personal experiences (i.e., environmental factors) can explain the differences in inheritance mode, age of onset, symptoms, and course of type I and type II alcoholism.

These two alcoholism subtypes, however, represent only the prototypes or extremes of a continuous spectrum of manifestations of alcoholism. Many of the subtype characteristics (e.g., personality traits) are inherited independently of each other, and all possible combinations of personality traits occur (Cloninger 1987*b*, Svrakic et al. 1993). Thus, extensive variability exists in the individual’s predisposition for alcohol abuse and related behaviors (Cloninger et al. 1995). Nevertheless, high harm avoidance and high novelty seeking appear to be the traits most strongly predisposing to type I and type II alcoholism, respectively.

The validity of this typology has been confirmed in numerous independent investigations, including studies of male and female twins in the United States (Pickens et al. 1991) and a replication of the original Stockholm adoption study. Although the replication study reproduced many of the findings of the original report, some discrepancies also existed. The resolution of these discrepancies will likely require further studies in additional subject populations.

## Figures and Tables

**Table 1:** Inheritance of the Susceptibility to Alcohol Abuse in Adoptees Participating in the Stockholm Adoption Study

Alcohol Abuse in the Biological Parents		Percentage of Adoptees With These Parents Who Abuse Alcohol	
	
Father	Mother	Sons	Daughters
			
No	No	14.7 %	2.8 %
Yes	No	22.4 %	3.5 %
No	Yes	26.0 %	10.3 %
Yes	Yes	33.3 %	9.1 %

SOURCE: Adapted from Sigvardsson et al. in press.

**Table 2:** Distinguishing Differences Between Type I and Type II Alcoholism^1^

Characteristic	Type I Alcoholism	Type II Alcoholism
Contributing factors	Genetic and environmental	Primarily genetic
Gender distribution	Affects both men and women	Affects men more often than women
Usual age of onset	After age 25	Before age 25
Common alcohol-related problems	Loss of control over drinking; binge drinking; guilt about drinking; progressive severity of alcohol abuse	Inability to abstain from alcohol; drinking frequently associated with fighting and arrests; severity of alcohol abuse usually not progressive
Characteristic personality traits	High harm avoidance and low novelty seeking; person drinks to relieve anxiety	High novelty seeking; person drinks to induce euphoria

1 The characteristics listed in this table define the type I and type II prototypes that only represent the two extremes of a continuous spectrum of manifestations of alcohol abuse.

## Footnotes

1 The use of the terms “alcoholism” and “alcohol abuse” frequently overlaps in the alcoholism literature; therefore, the terms are used in this article as they were used in the original studies cited.

2 In some of these studies, the researchers identified two alcoholic subtypes that corresponded to the type I and type II alcoholics in many characteristics but differed with respect to other characteristics.

3 High novelty seeking behavior is associated with disinhibition of prolactin release at rest. Dopamine activity, which is low in people who are high in novelty seeking, inhibits prolactin release.
